# Self-Reported Influence of Internal and External Resources and Class Attendance on Medical Students’ Pre-Clerkship Performance

**DOI:** 10.1007/s40670-025-02622-2

**Published:** 2026-01-03

**Authors:** Wenxin Yang, Benjamin T. Griffeth, William S. Wright

**Affiliations:** 1 University of South Carolina School of Medicine Greenville, Greenville, SC 29605 USA; 2 Prisma Health, Greenville, SC 29605 USA; 3 Department of Biomedical Sciences, University of South Carolina School of Medicine Greenville, Greenville, SC 29605 USA

**Keywords:** Curriculum, Resource utilization, Attendance, Medical student, Pre-clerkship performance

## Abstract

Medical students employ various learning strategies to master medical knowledge content. It remains uncertain whether these different approaches impact performance differently between female and male students. In a two-pass, predominately lecture-based biomedical science curriculum (pre-clerkship), end-of-course survey responses (related to resource utilization and attendance), video capture viewing time, and course performance were analyzed. Between females and males, there was a significant but modest difference in attendance, resources used, and viewing time in several courses with no difference in overall course grades. Our results indicate that use of various learning resources and attendance patterns are not detrimental to medical knowledge acquisition.

## Background

Medical students must navigate a vast and diverse body of knowledge while balancing the demands of course assessments and standardized exams, such as the USMLE^®^ Step 1. The decisions students make regarding study strategies—including reliance on internal (i.e. university generated) resources versus external (i.e. non-university generated) resources, in-person class attendance, and use of video capture technologies—may influence their overall academic performance. However, the extent of these influences remains unclear.

Only a few studies have compared the use of in-person, online, or video-recorded learning and outcomes in the graduate biomedical science courses with two showing better performance by students who attended class compared to video-recorded or hybrid (i.e. a course with some students attending in-person at the same time as students attending remotely) learning [1, 2] and with one in medical students showing no variance between students in synchronous online or asynchronous online learning [3]. There is a similar lack of data regarding gender-based variance in utilization of asynchronous learning and its outcomes [4–8] and none in medical students. One study found a negative effect on female-identified students using asynchronous learning; the authors posited that differences in interaction between course materials, increased home responsibilities, and outside work obligations may limit their capacity to learn [7]. Another study found that female students earning below the class average attended class less frequently than female students earning a grade above the class average while there was no difference in males [8]. With the National Center for Education Statistics reporting slightly more than half of students in undergraduate education enrolled for purely online class content [9], and more than 55% of medical school matriculants being women [10] further study of asynchronous learning’s effects—especially by gender—is needed.

This study evaluates the relationship between these factors in biomedical science pre-clerkship course outcomes at the University of South Carolina School of Medicine Greenville. By examining this relationship, we seek to provide insights that may guide the development of a more effective, learning-centered curriculum to support students. Therefore, this study aims to (1) assess the impact of internal (university generated) resources versus external (non-university generated) resources on course performance, (2) analyze the relationship between class attendance and course performance, (3) evaluate the role of video capture access time on course performance, and (4) examine gender differences in the study aims 1–3.

## Activity

### Design and Participants

This is a retrospective cohort study that analyzed data from pre-clerkship students at the University of South Carolina School of Medicine Greenville starting the first year of medical school between 2020 and 2022. Students self-assigned their gender identifications at time of enrollment. There were 348 total students with 203 (58%) female and 145 (42%) male students.

### Data Collection

Course sessions were recorded using the video platform Panopto^®^. This study analyzed the video capture viewing time per student within each course.

The end-of-course evaluation questions analyzed were: (1) For what percentage of in-class activities did you attend?, (2) For what percentage of module content did you rely on university generated resources?, and (3) For what percentage of module content did you rely on non-university generated resources?. Response options for each question were: 0–20%, 21–40%, 41–60%, 61–80%, and 81–100%. A point value was assigned for each response option: 1 for 0–20%, 2 for 21–40%, 3 for 41–60%, 4 for 61–80%, and 5 for 81–100% to calculate a pre-clerkship mean for responses to each question. Examples of university generated resources used by our students include video capture of synchronous sessions, PowerPoint slides created by our faculty, and recorded lectures. Examples of non-university generated resources used by our students include research articles, case reports and the UWorld question bank. University and non-university generated resources are used asynchronously. Responses to end-of-course evaluations are required and self-reported upon completion of each course. Response rates ranged between 93 and 99%. Students that did not complete end-of-course evaluations were reported to the Honor and Professionalism Committee for review per policy. Two missed evaluations resulted in a written warning from the promotions committee with escalating sanctions following three missed evaluations.

The video capture viewing time per student and end-of-course evaluation data were combined and de-identified for analysis.

### Statistical Analysis

Statistical analysis was performed with the software program International Business Machines^®^ (IBM) Statistical Package for the Social Sciences^®^ (SPSS) (v29.0.1.0). Pearson product-moment correlation was performed for pre-clerkship academic performance and pre-clerkship average video capture viewing time. Spearman rank-order correlation coefficient was performed to explore relationships between behaviors (i.e. course attendance, utilization of university generated resources, and non-university generated resources) and academic performance. An analysis of variance (ANOVA) was performed to determine differences between groups (i.e.: 0–20%, 21–40%, 41–60%, 61–80%, and 81–100%) in the following areas: (1) grades, (2) student video capture viewing time, (3) university generated resources, and (4) non-university generated resources. Independent T tests were performed to compare gender differences in the following areas for each course: (1) grades, (2) student video capture viewing time, (3) university generated resources, and (4) non-university generated resources. Statistical significance was set at *p* < 0.05.

### Ethics Approval

This study was reviewed and received exemption by the University of South Carolina Institutional Review Board.

## Results

### Final Course Grade

There was no difference in pre-clerkship biomedical science course grades or pre-clerkship weighted average between females and males (Table 1).

**Table 1:** Female and male comparison of final course grade and video capture viewing time, and end-of-course evaluation responses

		Final Course Grade				Video Capture Viewing Time (minutes)				For what percentage of in-class activities did you attend?				For what percentage of module content did you rely on university generated resources?				For what percentage of module content did you rely on non-university generated resources?			
Course Name	Gender	*N*	Mean	SEM	*p* value	*N*	Mean	SEM	*p* value	*N*	Mean	SEM	*p* value	*N*	Mean	SEM	*p* value	*N*	Mean	SEM	*p* value
Foundations	Male	139	86.25	0.70	0.118	140	1770	116	0.947	138	2.9	0.1	0.457	138	4.3	0.1	0.948	136	1.6	0.1	0.108
	Female	190	84.78	0.61		190	1761	96		189	3.0	0.1		189	4.3	0.1		188	1.4	0.1	
SF1	Male	139	86.19	0.68	0.071	141	2301	125	0.854	128	3.2	0.1	0.990	131	4.1	0.1	0.715	131	2.1	0.1	0.905
	Female	189	84.51	0.62		192	2330	99		186	3.2	0.1		186	4.2	0.1		185	2.1	0.1	
SF2	Male	137	86.74	0.62	0.669	139	2005	107	0.279	134	2.9	0.1	0.721	134	4.2	0.1	0.654	134	2.1	0.1	0.594
	Female	187	86.39	0.53		189	2150	79		186	3.0	0.1		186	4.2	0.1		186	2.2	0.1	
Neuro	Male	136	90.11	0.69	0.750	136	1391	83	0.339	133	2.8	0.1	0.499	134	3.9	0.1	0.470	134	2.5	0.1	0.299
	Female	187	90.37	0.48		187	1494	69		181	2.7	0.1		182	4.0	0.1		182	2.3	0.1	
Defenses	Male	136	88.14	0.77	0.287	136	1660	97	0.005**	132	2.7	0.2	0.018*	132	4.2	0.1	0.165	132	2.0	0.1	0.037*
	Female	185	87.09	0.63		185	2018	82		178	2.3	0.1		179	4.4	0.1		179	1.7	0.1	
BPIDT	Male	139	84.54	0.82	0.148	139	1530	86	0.012*	133	2.1	0.1	0.047*	134	3.3	0.1	0.192	133	3.9	0.1	0.073
	Female	188	83.02	0.66		188	1809	68		182	1.8	0.1		182	3.5	0.1		184	3.6	0.1	
HemOnc	Male	139	84.63	1.00	0.635	139	1460	72	0.015*	133	2.2	0.1	0.350	135	3.9	0.1	0.074	135	2.9	0.1	0.062
	Female	187	85.22	0.80		187	1707	68		179	2.1	0.1		179	4.1	0.1		178	2.6	0.1	
MBB	Male	139	85.90	0.70	0.881	139	1772	79	0.215	136	2.3	0.1	0.118	136	4.0	0.1	0.448	134	2.8	0.1	0.040*
	Female	185	86.03	0.55		187	1903	70		178	2.0	0.1		180	4.1	0.1		179	2.5	0.1	
CPR	Male	138	85.36	0.70	0.099	136	2714	127	0.061	129	2.4	0.1	0.017*	129	3.8	0.1	0.044*	129	3.0	0.1	0.949
	Female	185	83.79	0.63		184	3026	107		176	1.9	0.1		177	4.1	0.1		176	3.0	0.1	
GI	Male	139	86.29	0.68	0.510	136	1282	58	0.216	130	2.0	0.1	0.071	130	4.0	0.1	0.191	129	3.0	0.1	0.423
	Female	184	85.67	0.62		179	1377	50		170	1.7	0.1		170	4.2	0.1		169	2.8	0.1	
EndoRepro	Male	136	85.62	0.73	0.478	132	1456	67	0.086	122	2.0	0.1	0.082	122	4.0	0.1	0.270	122	3.0	0.1	0.178
	Female	180	84.93	0.65		174	1613	60		158	1.7	0.1		160	4.1	0.1		160	2.7	0.1	
MDR	Male	136	89.09	0.69	0.971	132	1075	52	0.156	121	2.0	0.1	0.339	121	4.0	0.1	0.497	121	3.0	0.1	0.058
	Female	178	89.12	0.58		173	1172	44		162	1.8	0.1		162	4.1	0.1		163	2.7	0.1	
≠†Pre-Clerkship Average	Male	130	87.02	0.59	0.679	130	1722	73	0.081	130	2.4	0.1	0.402	130	4.0	0.1	0.118	130	2.6	0.1	0.200
	Female	167	86.71	0.47		167	1889	62		167	2.3	0.1		167	4.1	0.1		167	2.5	0.1	

Legend: *p<0.05, **p<0.01 (2-tailed). *N* Number of students, *SEM* Standard Error of the Mean. ≠Includes data for students completed all courses in the pre-clerkship curriculum. †Pre-clerkship weighted average for “Final Course Grade”. Courses: *Foundations * Molecular and Cellular Foundations of Medicine, *SF1* Structure and Function of the Human Body 1, *SF2* Structure and Function of the Human Body 2, *Neuro* Neuroscience, *Defenses* Defenses and Responses, *BPIDT * Biomedical Principles of Disease and Therapy, *HemOnc* Hematology/Oncology Systems, *MBB* Mind, Brain, and Behavior, *CPR* Cardiovascular/Pulmonary/Renal Systems, *GI* Gastrointestinal/Hepatic Systems, *EndoRepro* Endocrine/Reproduction Systems, *MDR * Musculoskeletal/Dermatology/Rheumatology Systems

### Class Attendance

**Fig. 1:**
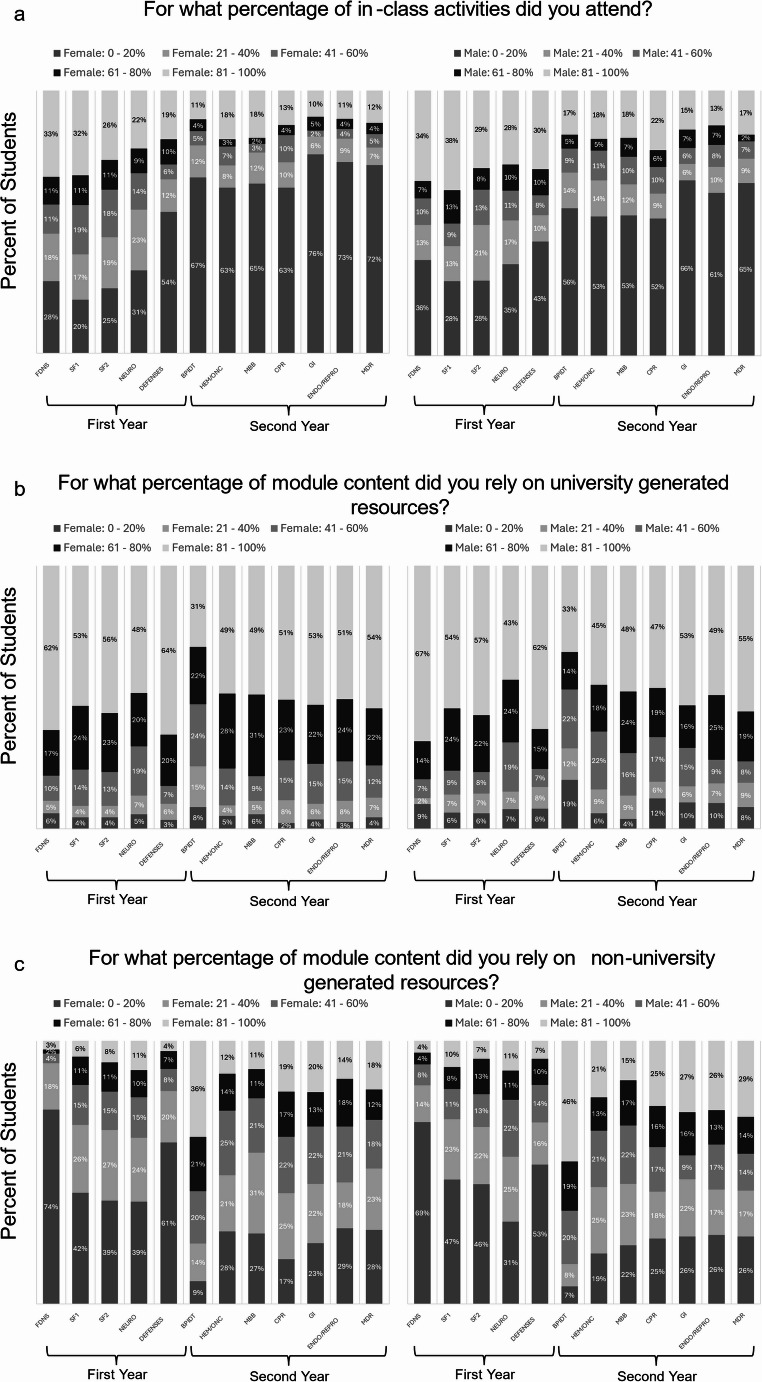
Female and male comparison of end-of-course evaluation question responses. Legend: N for Females ranged 162–189. N for Males ranged 121–138. N counts varied based on response rate per course with response rates ranging between 93–99%. 1a) Percentage of students responding to percentage of in-class activities attended. 1b) Percentage of students responding to percentage of university generated resources relied on for module content. 1c) Percentage of students responding to percentage of non-university generated resources relied on for module content. Courses: Foundations = Molecular and Cellular Foundations of Medicine; SF1 = Structure and Function of the Human Body 1; SF2 = Structure and Function of the Human Body 2; Neuro = Neuroscience; Defenses = Defenses and Responses; BPIDT = Biomedical Principles of Disease and Therapy; HemOnc = Hematology/Oncology Systems; MBB = Mind, Brain, and Behavior; CPR = Cardiovascular/Pulmonary/Renal Systems; GI = Gastrointestinal/Hepatic Systems; EndoRepro = Endocrine/Reproduction Systems; MDR = Musculoskeletal/Dermatology/Rheumatology Systems

There was a significant difference in attendance between females and males for three of the twelve biomedical science courses with females having lower attendance compared to males (Table 1). There was no correlation between pre-clerkship weighted average and pre-clerkship class attendance average for females (*r* = 0.000, *p* = 0.995, 95% *CI* [−0.153, 0.154], *N* = 174) and males (*r* = 0.044, *p* = 0.612, 95% *CI* [−0.131, 0.216], *N* = 135). The percent of female students reporting attending 0–20% of in-class activities rose above 50% for the final course of the first year. The percent of male students reporting attending 0–20% of in-class activities rose above 50% starting in the first course of the second year. The decrease in course attendance was sustained through the end of the second year for female and male students (Fig. 1a).

### Video Capture Viewing Time

There was a significant difference in video capture viewing time between females and males for three of the twelve biomedical science courses with females having higher viewing times compared to males (Table 1). There was no correlation between pre-clerkship weighted average and pre-clerkship video capture viewing time average across all courses for females (*r* = 0.064, *p* = 0.399, 95% *CI* [−0.085, 0.211], *N* = 174) and males (*r* = 0.018, *p* = 0.834, 95% *CI* [−0.151, 0.187], *N* = 135).

### Internal (University Generated) Resource Utilization

There was a significant difference in university resources used between females and males for only one of the twelve biomedical science courses with females using more university resources compared to males (Table 1). There was no correlation between pre-clerkship weighted average and pre-clerkship university resource utilization average for females (*r* = −0.045, *p* = 0.555, 95% *CI* [−0.197, 0.109], *N* = 174) and males (*r* = −0.092, *p* = 0.290, 95% *CI* [−0.261, 0.084], *N* = 135).

#### External (Non-University Generated) Resource Utilization

There was a significant difference in university resources used between females and males for two of the twelve biomedical science courses with females using less non-university resources compared to males (Table 1). There was no correlation between pre-clerkship weighted average and pre-clerkship non-university resource utilization average for females (*r* = −0.028, *p* = 0.713, 95% *CI* [−0.180, 0.126], *N* = 174) and males (*r* = −0.027, *p* = 0.757, 95% *CI* [−0.200, 0.148], *N* = 135). The number for female and male students relying on non-university generated resources for 0–20% of module content was highest in the first pre-clerkship course (74% of females and 69% of males) with the percent of students being > 40% for all first-year courses. In the second year, the number for female and male students relying on non-university generated resources for 0–20% of module content decreased to values similar to students relying on non-university generated resources for 81–100% of module content (Fig. 1c). Though the students were allowed to name external resources used, there were no qualitative differences in platforms used when compared between modules.

## Discussion

Our study provides valuable insights into the relationship between academic performance and various factors such as external resource utilization and classroom attendance of pre-clerkship medical students at our institution.

Declining class attendance as students’ progress through the curriculum does not seem to affect performance for female or male students, suggesting that alternative learning methods may be equally effective. Additionally, there is no difference in pre-clerkship weighted average with higher video capture viewing time; this point is noteworthy due to the trend of females to use video capture at a higher rate than males (*p* = 0.081). These findings are in direct contrast to prior publications that show female students do not perform well when learning outside of the traditional classroom [7, 8]. It is doubtful that this is due to variance in the curriculum presented but more likely due to comfort with video capture as a primary source of data acquisition.

These findings highlight the need for a balanced, flexible curriculum that accommodates diverse study strategies while emphasizing the importance of meeting the needs of students attending in the classroom, using video capture as well as those using both university-generated and non-university generated resources. As the medical education landscape evolves, tailoring medical education, especially within the pre-clerkship phase, to the needs of each student could enhance student success and better support their preparation for coursework.

Some limitations to our study include data from a single institution and reliance on student-reported data for end-of-course evaluation responses. We also do not have data for reasons why students either chose to attend or not attend class and why students select certain resources to gain knowledge acquisition. Finally, the student survey data does not ask them to rate the impact of live attendance versus video capture nor the impact of university versus non-university resources.

## Conclusion

Our data show that student performance in the pre-clerkship biomedical science courses is not different between females and males despite differences in use of video capture technology versus attendance in some pre-clerkship courses. We show there is a decrease in class attendance and an increase in non-university generated resources for females and males as students progress through the pre-clerkship curriculum.

## Data Availability

The datasets used and/or analyzed during the current study are available from the corresponding author on reasonable request.
